# Comparison of different telerehabilitation protocols for urogenital symptoms in females with multiple sclerosis: a randomized controlled trial

**DOI:** 10.1007/s10072-024-07742-y

**Published:** 2024-09-03

**Authors:** Manuela Deodato, Mia Fornasaro, Miriam Martini, Francesca Zelesnich, Arianna Sartori, Alessandra Galmonte, Alex Buoite Stella, Paolo Manganotti

**Affiliations:** 1 School of Physiotherapy, Department of Medicine, Surgery and Health Sciences, University of Trieste, via Pascoli 31, 34100 Trieste, Italy; 2 PhD program in Personalized Medicine and Innovative Therapies, Department of Medicine, Surgery and Health Sciences, University of Trieste, Strada di Fiume 447, 34149 Trieste, Italy; 3 Clinical Unit of Neurology, Department of Medicine, Surgery and Health Sciences, Cattinara University Hospital (ASUGI), University of Trieste, Strada di Fiume 447, 34149 Trieste, Italy

**Keywords:** Incontinence, Sexual dysfunction, Physiotherapy, pelvic floor training, Telemedicine

## Abstract

Telerehabilitation has been suggested to be equally effective than in-person rehabilitation, and could be helpful to increase participation and reduce barriers. People with multiple sclerosis (MS) often present urogenital dysfunctions, impairing independence and quality of life (QoL). Since the different available telerehabilitation protocols, the present study aimed to compare a live video urogenital rehabilitation intervention protocol (REMOTE) with a home-based pre-recorded video protocol (SELF). A randomized-controlled trial was performed, with 14 females with MS being allocated in the REMOTE group (36 ± 9 y) and 14 females in the SELF group (37 ± 7 y). Both telerehabilitation protocols were identical in terms of contents (including pelvic floor training and relaxation exercises), frequency and duration, consisting of 10 sessions of 45 min each, every 5 days. Questionnaires were administered at the beginning and the end of the study: Short Form Health Survey 36 (SF-36), Beck Depression Inventory (BDI), Female Sexual Function Index (FSFI), International Consultation on Incontinence Questionnaire (ICIQ) symptoms and related QoL, the main outcome being ICIQ incontinence score. Despite most of the outcomes improved in both groups, REMOTE was found to be more effective than SELF in most of the SF-36 domains (from *p* < 0.001 pη^2^ 0.555 to *p* = 0.044 pη^2^ 0.147), FSFI (*p* = 0.001 pη^2^ 0.373), ICIQ (*p* = 0.003 pη^2^ 0.291). Despite the home-based pre-recorded videos could be effective in improving urogenital symptoms, live video urogenital rehabilitation results in larger improvements. Telerehabilitation should be encouraged for urogenital dysfunctions in females with MS, and pre-recorded videos could represent an alternative when live sessions are not available.

**Clinical trial registration** This randomized controlled trial was registered on ClinicalTrials.gov with the number NCT05984095.

**Supplementary Information:**

The online version contains supplementary material available at 10.1007/s10072-024-07742-y.

## Introduction

Multiple Sclerosis (MS) is characterized by a plethora of symptoms, such as numbness, tingling, weakness, vision loss, gait impairment, incoordination, imbalance, and bladder dysfunction, as well as fatigue and heat sensitivity [1, 2]. Bladder dysfunction represents one of the main autonomic symptoms in MS, with a prevalence in females between 48 and 80%, and is characterized by urological manifestations [3]. Among these symptoms, incontinence (i.e., the failure to store urine) and retention (i.e., the failure to empty the bladder) influence the quality of life (QoL) of people with MS (pwMS), as they affect overall well-being and self-esteem [4]. In addition, sexual dysfunction is also reported in females with MS, with a prevalence of 61% and the odds of developing sexual dysfunction in comparison with controls being 3.05 [5], with main symptoms including diminished desire, arousal/erectile dysfunction, and orgasmic/ejaculatory dysfunction [6]. Sexual dysfunctions have been reported to be often underdiagnosed and undertreated, while being associated with depression, and reduced quality of life, and may have broader implications related to relationships, fertility, pregnancy, and parenting [7].

A multidisciplinary approach is recommended to treat urogenital symptoms, including pharmacological and non-pharmacological therapies, with rehabilitation representing a valid approach to improve symptoms through pelvic floor muscle training (PFMT), relaxation techniques and better control over body functions [4, 8, 9]. In general, structured, multidisciplinary rehabilitation programs and physical therapy (exercise or physical activities) can improve functional outcomes (mobility, muscle strength, aerobic capacity), and quality of life [10]. However, some motivational factors and barriers might be present, limiting the participation of pwMS in rehabilitation programs, including fatigue, heat, lack of support and advice, and impairments arising from the condition and time [11, 12]. Telemedicine represents one possible solution to support and provide the healthcare services in pwMS [13–15], with telerehabilitation representing one of the only possibilities to propose physiotherapy interventions to people living in remote areas [16], or like during the recent lockdown [17], telerehabilitation might also represent a facilitator for participation in physiotherapy programs, by reducing travel time and cost [18]. The effectiveness of the telerehabilitation protocols has been suggested to be comparable with in-person rehabilitation or better than no rehabilitation [19]. Regarding telerehabilitation for urogenital symptoms, it has been suggested that it might be feasible and acceptable, although more studies are needed to identify the best protocols [20].

The present study aimed to evaluate the efficacy of a remotely-supervised telerehabilitation protocol (REMOTE) compared to a video-based self-telerehabilitation protocol (SELF), on measures of bladder and sexual symptoms, depression, and QoL, and in particular incontinence, in females with MS.

## Methods

A randomized controlled, two-arm, assessor-blinded study was performed from September to November 2023 in a University Hospital setting, within the School of Physiotherapy program. Participants were recruited among females referring to the University Hospital Multiple Sclerosis Center and in collaboration with patient associations. Inclusion criteria were: females with a diagnosis of relapsing-remitting MS confirmed by a neurologist and based on the McDonald criteria [21] from not less than 1 y, aged between 18 and 50 years, Expanded Disability Status Scale (EDSS) < 4.5, self-reported symptoms of urinary incontinence. Participants were excluded if they reported being in menopause, previous history of bladder or urogynaecological surgery, previous history of major abdominal surgery, had delivery < 6 months from the start of the study, had body mass index (BMI) > 30, or if they participated to rehabilitation protocols in the previous year. Based on previous studies [20], assuming an effect size (ES) *d* = 1.11, a power of 0.80, an α error probability of 0.05, a priori sample size was calculated as 14 using G*Power (ver. 3.1, University of Kiel, Kiel, Germany). The clinical trial was prospectively registered on ClinicalTrials.gov (NCT05984095) and followed the Consolidated Standards of Reporting Trials [22]. Ethical approval was obtained from the local ethical committee (007_2021), written informed consent was obtained from the participants volunteering for the study, and all the procedures were performed according to the principles of the Declaration of Helsinki.

After verification of the inclusion and exclusion criteria, participants who volunteered for the study signed the informed consent and were randomized to one of the two telerehabilitation groups: REMOTE or SELF. To reduce the risk of bias due to knowledge of allocation, one of the investigators performed the randomization procedure by tossing a coin for each participant (AS), one investigator administered and collected the questionnaires (MD) and two investigators performed the statistical analyses and visualization of the results (MM and ABS). The same physiotherapist (MF) delivered the telerehabilitation protocol.

### Telerehabilitation protocols

Two telerehabilitation protocols were designed based on previous literature with a consensus between physiotherapists, clinical exercise physiologists and neurologists with experience in MS and urogenital rehabilitation [20]. Both telerehabilitation protocols were similar in terms of number of sessions (10 in total), frequency (once every 5 days), duration (45 min), and content. The REMOTE group performed the physiotherapy sessions in a telerehabilitation fashion through a one-to-one video call on the phone or tablet, using Skype (ver. 15.75.140.0, Microsoft Inc, Washington) according to a previous protocol [20], and the exercises were shown and monitored remotely by the physiotherapist. Both cameras were open during the entire training, and both the physiotherapist and the participant wore comfortable clothes that allowed their body movements to be well visible. In the SELF group, the exercises were available in pre-recorded videos and the participants were allowed to access it when and where they preferred, respecting the requested frequency. No supervision was provided during the training, but the participants were asked to confirm they performed the training to the physiotherapist. Each session consisted of different exercises, progressive in terms of intensity and volume, including a multidomain physiotherapy protocol based on previous literature [8, 23–31]: (i) breathing exercises, (ii) PFMT, (iii) proprioceptive exercises, (iv) motor coordination and balance, (v) functional training, vii) and body scan technique. A complete description of the proposed exercises is provided in Supplementary File 1. For each exercise, the physiotherapist showed it clearly and asked the participant to reproduce it. Only in the REMOTE group, did the physiotherapist provide constant feedback. Both groups received an initial educational intervention on symptoms management and a healthy lifestyle [32]. To enter the final analysis, all the participants had to participate in at least 9 sessions.

### Outcomes assessment

All the assessments were performed at the start (one week before) and at the end (one week after) of the protocol, completing 6 questionnaires regarding pain, quality of life and health. Depression symptoms were evaluated with the Beck Depression Inventory scale (BDI-II, 0–63, the higher the score worse the depression, < 14 suggesting absent or mild depressive symptoms) [33]. Perceived quality of life was assessed with the Short Form Health Survey 36 (SF-36, 0-100 for each domain, the higher the score better the quality of life) The domains are: physical function (PF), role limitation due to physical problems (RP), bodily pain (BP), general health (GH), vitality (VT), social functioning (SF), role limitation due to emotional problems (RE), and mental health (MH) [34]. Since pain was assessed in the SF-36 questionnaire, the numeric rating scale (NRS) - initially considered in the protocol - was not administered. Perceived sexual health was evaluated with the female sexual function index (FSFI, 1–5 for each item, greater score indicating greater levels of sexual functioning, 2–36 total score) [35]. Finally, urinary incontinence symptoms were assessed with the International Consultation on Incontinence Questionnaire (ICIQ, 0–21, the higher the score worse the symptoms) [36]. In addition, ICIQ-FLUTS (Female Lower Urinary Tract Symptoms) with three domains: 0–16 filling symptoms subscale, 0–12 voiding symptoms subscale, 0–20 incontinence symptoms subscale, higher the score worse the symptoms, and ICIQ-LUTSqol (Lower Urinary Tract Symptoms - Quality of Life, 19–76 overall score with greater values indicating increased impact on quality of life) [36, 37]. ICIQ-FLUTS incontinence was considered the main outcome of the present study.

### Statistical analysis

This is the first analysis of these data. All data were analyzed with SPSS v.23 (IMB inc.) and a graphical representation of the results was performed with GraphPad Prism. Since all the participants completed the protocol and assessments, no intention-to-treat analysis was necessary. Kolgomorov-Smirnov test was used to assess the normal distribution of the results. All data were normally distributed. Data are reported as mean and standard deviation. Independent-sample t-test and chi-square test were performed to compare demographics and clinical characteristics at the baseline. A mixed-factors analysis of variance (ANOVA) was performed (between groups: REMOTE vs. SELF, within groups: pre vs. post). In the case of significant interaction, simple main effects were reported. Post-hoc analysis was performed with Sidak’s correction. ANOVA’s effect size was reported as pη^2^, and significance was set for *p* < 0.05.

## Results

**Fig. 1:**
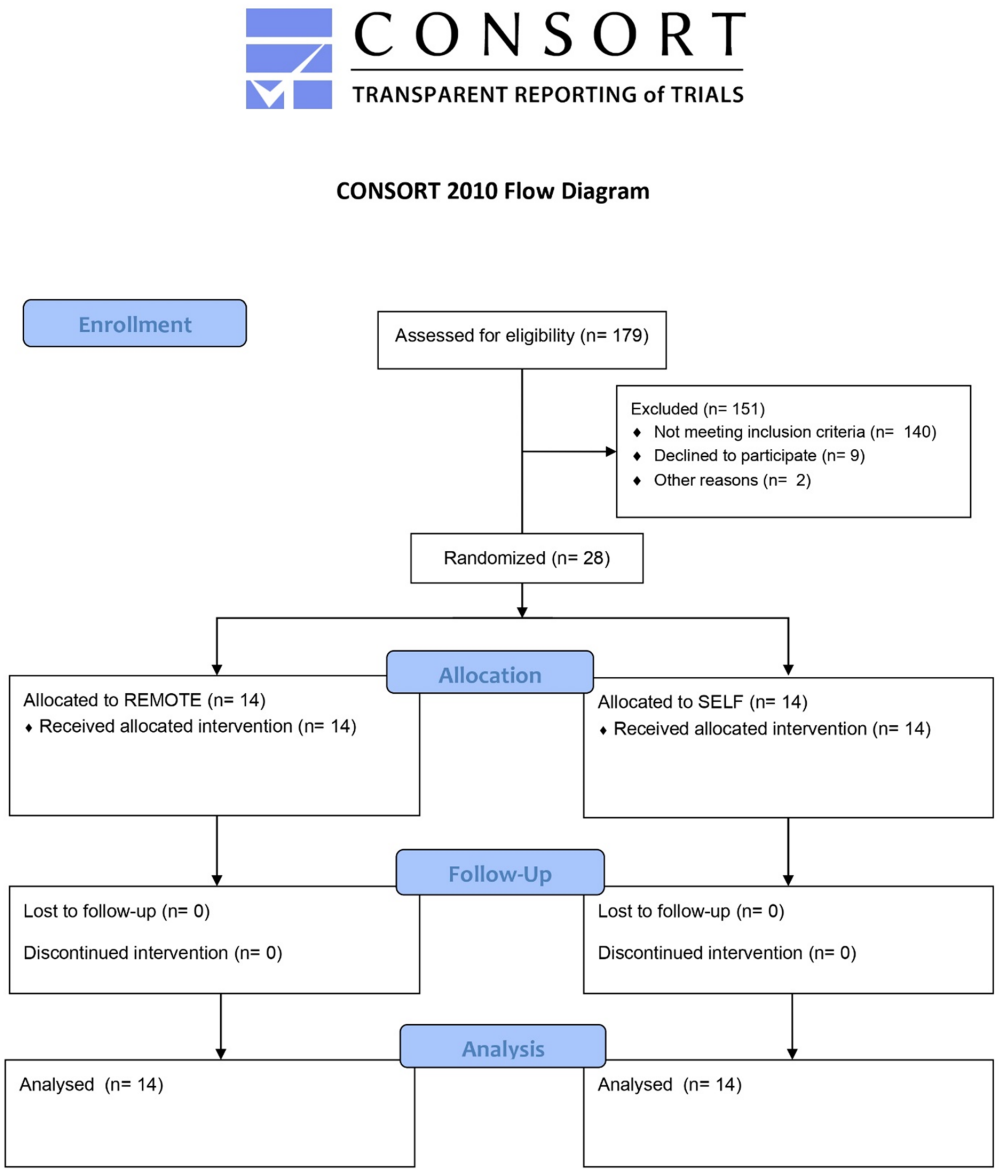
CONSORT 2010 flow diagram

All the participants completed the study protocol and assessments, and no missing data were present. The CONSORT 2010 Flow Diagram is reported in Fig. 1. The demographics and clinical characteristics of the sample are reported in Table 1.

**Table 1:** Demographics and clinical characteristics of the sample. Data presented as means ± standard deviations

	REMOTE *n* = 14	SELF *n* = 14	Significance
Age (years) Body mass (kg) Body height (m) BMI (kg/m2) EDSS Disease duration (years) Childbirth (n) Education (years)	36 ± 9 61.3 ± 7.6 1.72 ± 0.07 20.7 ± 1.5 1 (1–2) 8.8 ± 5.2 10 13 ± 3	37 ± 7 66.9 ± 8.3 1.74 ± 0.07 22.1 ± 1.7 2 (1–2) 8.9 ± 4.2 6 13 ± 4	0.886 0.072 0.496 **0.026** 0.167 0.968 0.127 0.911

**Notes**: Body Mass Index (BMI), Expanded Disability Status Scale (EDSS), significance at the indepenent-samples t test, Mann-Whitney U Test, and chi-square test, *p* < 0.05

A significant group x time interaction was reported for most of the SF-36 domains: PF (F_1,26_ = 34.400, *p* < 0.001, pη^2^ = 0.555), RP (F_1,26_ = 4.680, *p* = 0.040, pη^2^ = 0.153), VT (F_1,26_ = 4.493, *p* = 0.044, pη^2^ = 0.147), SF (F_1,26_ = 10.077, *p* = 0.004, pη^2^ = 0.279), MH (F_1,26_= 17.349, *p* < 0.001, pη^2^ = 0.400). For the remaining domains, a significant time effect was found for BP (F_1,26_= 24.356, *p* < 0.001, pη^2^ = 0.522), GH (F_1,26_= 20.968, *p* < 0.001, pη^2^ = 0.446) and RE (F_1,26_= 28.448, *p* < 0.001, pη^2^ = 0.522).

A significant group x time interaction was also found in the FSFI (F_1,26_= 15.436, *p* = 0.001, pη^2^ = 0.373), ICIQ (F_1,26_= 10.662, *p* = 0.003, pη^2^ = 0.291), ICIQ-FLUTS incontinence (F_1,26_= 12.745, *p* = 0.001, pη^2^ = 0.329), and ICIQ-LUTSqol (F_1,26_= 41.529, *p* < 0.001, pη^2^ = 0.615). A significant time effect was found for BDI (F_1,26_= 25.373, *p* < 0.001, pη^2^ = 0.494) and ICIQ-FLUTS filling (F_1,26_= 13.444, *p* = 0.001, pη^2^ = 0.341). In particular, within the 6 participants with BDI score ≥ 14 in the REMOTE group before the intervention, 2 of them reduced the score below the cut-off value after the intervention. In contrast, 7 participants in the SELF group reported a BDI score ≥ 14 both before and after the intervention. No group differences were present in any of the outcomes.

**Fig. 2:**
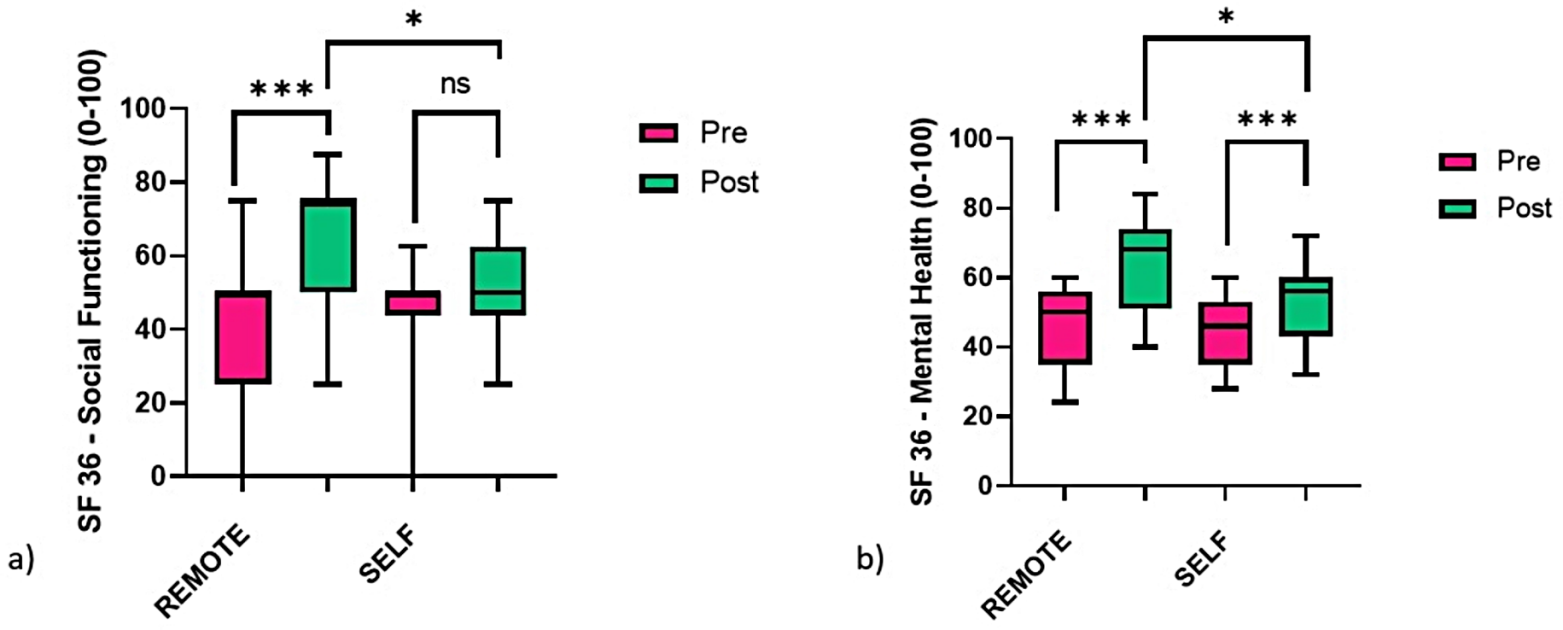
Boxplots comparing the pre (green) and post (pink) scores of SF-36 (**a**) social functioning (SF) and (**b**) mental health (MH) between the live video physiotherapy (REMOTE, *n* = 14) and pre-recorded video physiotherapy (SELF, *n* = 14) telerehabilitation groups. A mixed-factors analysis of variance (ANOVA) was performed, significance reported for simple main effects, * *p* < 0.05, ** *p* < 0.01, *** *p* < 0.001

**Fig. 3:**
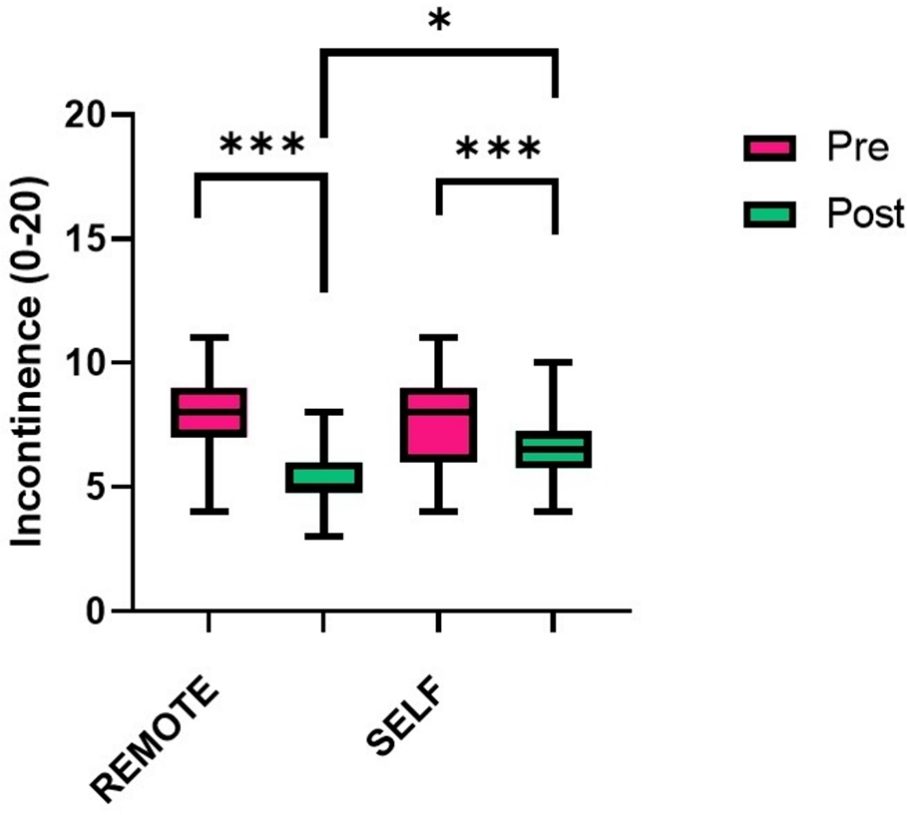
Boxplots comparing the pre (green) and post (pink) scores of ICIQ-FLUTS incontinence between the live video physiotherapy (REMOTE, *n* = 14) and pre-recorded video physiotherapy (SELF, *n* = 14) telerehabilitation groups. A mixed-factors analysis of variance (ANOVA) was performed, significance reported for simple main effects, * *p* < 0.05, ** *p* < 0.01, *** *p* < 0.001

As reported in Table 2, most of the variables were found to statistically improve after the telerehabilitation intervention in both groups, with larger improvements in the REMOTE group compared to SELF: a significant difference was found post-physiotherapy, REMOTE presenting greater scores than SELF for SF (13.393, 95% CI: 0.346–26.440, *p* = 0.045) and MH (12.286, 95% CI: 2.457–22.114, *p* = 0.016) (Fig. 2) and a lower score for incontinency (-1.429, 95% CI: -2.523 – − 0.334, *p* = 0.012) (Fig. 3).

**Table 2:** Outcomes before and after the telerehabilitation intervention. Data presented as means ± standard deviations

	REMOTE *n* = 14		SELF *n* = 14		Significance
	pre	post	pre	post	*Group x Time (*pη²*)*
*SF-36 (0-100)*					
PF RP BP GH VT SF RE MH BDI (0–63) FSFI (2–36) ICIQ (0–21) *ICIQ-FLUTS* Filling (0–16) Voiding (0–12) Incontinence (0–20) ICIQ-LUTSqol (19–76)	67.5 ± 11.7b 23.2 ± 22.9b 45.9 ± 18.9b 51.1 ± 13.6b 37.5 ± 13.6b 41.1 ± 18.6b 23.8 ± 30.4b 46.3 ± 12.3b 13.1 ± 6.4b 19.8 ± 2.4b 11.6 ± 2.1b 2.6 ± 1.2b 1.1 ± 1.0 7.8 ± 1.7b 42.2 ± 7.9b	77.9 ± 11.2b 44.6 ± 28.0b 55.5 ± 18.0b 56.4 ± 16.6b 42.1 ± 16.7b 63.4 ± 18.0a, b 50.0 ± 31.3b 65.4 ± 13.8a, b 11.5 ± 5.4b 22.2 ± 1.8b 8.3 ± 1.7b 2.1 ± 0.9b 0.9 ± 0.7 5.2 ± 1.2a, b 30.4 ± 7.9b	66.7 ± 8.9b 19.6 ± 22.3b 48.0 ± 15.2b 45.4 ± 14.2b 39.6 ± 7.2 43.7 ± 16.1 16.7 ± 21.7b 44.9 ± 10.1b 15.4 ± 5.6b 20.0 ± 1.7b 11.1 ± 2.7b 2.3 ± 1.2 1.0 ± 0.9 7.8 ± 1.9b 39.1 ± 6.8b	71.8 ± 10.1b 30.4 ± 22.3b 53.2 ± 15.4b 50.7 ± 14.7b 39.6 ± 11.0 50.0 ± 15.5a 35.7 ± 20.5b 53.1 ± 11.4a, b 14.4 ± 5.6b 20.9 ± 1.9b 9.4 ± 2.1b 2.0 ± 1.1 0.8 ± 0.4 6.6 ± 1.6a, b 34.6 ± 5.2b	**<0.001 (0.555)** **0.040 (0.153)** 0.149 (0.078) > 0.999 (< 0.001) **0.044 (0.147)** **0.004 (0.279)** 0.407 (0.027) **< 0.001 (0.400)** 0.273 (0.046) **0.001 (0.373)** **0.003 (0.291)** 0.327 (0.037) 0.764 (0.004) **0.001 (0.329)** **< 0.001 (0.615)**

**Notes**: Short Form Health Survey 36 (SF-36, 0-100 for each domain, higher the score better the quality of life): physical function (PF), role limitation due to physical problems (RP), bodily pain (BP), general health (GH), vitality (VT), social functioning (SF), role limitation due to emotional problems (RE), and mental health (MH). Beck Depression Inventory scale (BDI, 0–63, higher the score worse the depression). Female sexual function index (FSFI, 1–5 for each item, greater score indicating greater levels of sexual functioning, 2–36 total score). International Consultation on Incontinence Questionnaire (ICIQ, 0–21, higher the score worse the symptoms) FLUTS (Female Lower Urinary Tract Symptoms, 0–16 filling symptoms subscale, 0–12 voiding symptoms subscale, 0–20 incontinence symptoms subscale, higher the score worse the symptoms), and LUTSqol (Lower Urinary Tract Symptoms - Quality of Life, 19–76 overall score with greater values indicating increased impact on quality of life). Mixed factors analysis of variance (ANOVA), interaction group x time (effect size, pη^2^); post-hoc for ^a^ significant difference between REMOTE and SELF at the same time, ^b^ significance difference between pre and post for each group, *p* < 0.05

## Discussion

The results of this study suggest that telerehabilitation might provide beneficial effects in females with MS, improving most of the outcomes associated with urogenital symptoms. Indeed, except for the ICIQ-FLUTS voiding symptom, a significant time effect was found, suggesting that both protocols improved the assessed symptoms. Nevertheless, the main outcome of the study ICIQ-LUTS incontinence, as well as most of the SF-36 and lower urinary tract and sexual dysfunction scales, were found to have a greater improvement in the group who received the supervised telerehabilitation protocol compared to those who received the video-based telerehabilitation protocol. Among the items with smaller differences between the two groups improvements, body pain, general health and role limitation due to emotional problems, as well as depression and bladder-filling symptoms, were found. Compared to a previous study, these findings show some similar and conflicting results; indeed, despite urogenital symptoms improvement was found after telerehabilitation, the authors reported that urinary incontinence severity, sexual dysfunction, QoL, depression, and anxiety symptom scores did not improve compared to a control group [20]. It should be reported that the telerehabilitation protocol was different compared to the one from the current study, as well as statistical analyses. In particular, during the REMOTE and SELF telerehabilitation protocol, PFMT was accompanied by whole-body exercises and relaxation techniques. In addition, the sample was characterized by a higher EDSS score than participants in the present study. As such, it could be speculated that telerehabilitation might be more effective for females with less severe disability.

Both the telerehabilitation protocols proposed in the present study (REMOTE and SELF) were well-accepted by the participants, as no drop-outs occurred and all the participants who completed the protocol participated in all the proposed training sessions. In particular, no differences in terms of adherence to the protocol were found in the SELF group, suggesting that the proposed protocol was effective in maintaining therapeutic compliance. Unsupervised pelvic floor rehabilitation has been suggested to be less effective than supervised training, and this might depend both on adherence and correct performance of the exercises [38]. However, some conflicting results have been reported, as previous research suggested no differences in terms of adherence and number of leakages between PFMT program with or without physiotherapist guidance in people with multiple sclerosis [39]. In the present study, bladder symptoms were found to improve in both supervised and unsupervised telerehabilitation groups; however, the incontinence symptom and incontinence-related QoL improved more during the supervised protocol. In contrast, the voiding symptom was found to be the only urinary symptom that did not present a significant improvement in either of the protocols. This might be possibly explained by the low score of the baseline ICIQ filling and voiding scales, indicating mild urinary dysfunction in the present population.

The improvement in sexual function that was reported after both telerehabilitation protocols is in line with previous findings suggesting that PFMT might improve these symptoms [40–42]. In particular the FSFI was used as an outcome of improvement, and most interventions resulted in an improvement although not superior to the proposed cut-off value of 26.5 to differentiate between women with and without sexual dysfunction [43]. In this study, all participants were characterized by a FSFI score suggestive of sexual dysfunction, and both interventions resulted in a significant improvement although not sufficient to exceed the cut-off score. However, to the best of the authors’ knowledge, this study is the first one suggesting that telerehabilitation might also improve sexual function in females with MS in line with standard physiotherapy protocols, and such improvements could be larger when supervised training was proposed compared to unsupervised training.

In terms of general QoL and depression symptoms, all items presented a significant improvement after both telerehabilitation protocols. Considering the SF-36 domains, most of them improved more after the supervised training compared to the unsupervised protocol; however, pain, role limitation due to emotional problems, general health, and BDI (depression) showed no significant differences between groups, suggesting that such aspects might be less dependent on the physiotherapist supervision. If a previous study found that anxiety and depression improve after PFMT training [44], prior telerehabilitation protocols did not report significant changes compared to a control group. The significant improvements reported after the REMOTE and SELF protocols in these items might be explained by the training protocol including breathing and relaxation techniques. In particular, body scan/mindfulness has been reported to be effective in improving depressive symptoms in pwMS [45].

Taken together, the findings from this study suggest that telerehabilitation could be effective in improving symptoms of females with MS reporting urogenital disorders, although training with a physiotherapist, despite remotely, could provide larger benefits, including a larger incontinence score improvement. Nonetheless, the SELF protocol (i.e., pre-recorded video-based physical therapy) showed also significant improvements in most of the outcomes and could help reach a larger number of individuals. Despite the lack of physical contact with a physical therapist remains a limitation [46], video-based physical therapy has been considered an effective way of rehabilitation [19]. Although home-based video exercise might be less effective than live video sessions with a physiotherapist, future studies should consider the advantages provided by a convenience, adherence, and cost-effectiveness analysis, in order to promote physical activity and rehabilitation in pwMS, reducing barriers and restrictions.

### Limitations and future perspectives

This study presents some limitations that should be acknowledged for a proper interpretation and generalization of the present results. First, the moderate sample size, despite being based on previous research (although with a slightly different design) and feasibility considering the recruitable population, with an a posteriori analysis on ICIQ reporting achieved power > 0.95. In addition, the sample was characterized by females with MS presenting low EDSS scores and most of the proposed scales did not indicate the presence of severe symptoms, therefore limiting generalization also due to the lack of data regarding minimum annual income. Second, the absence of a control group not receiving any physiotherapy intervention does not allow to exclude any effect depending on the time course of the study or repetition of the outcomes’ assessment. Third, adherence to the SELF protocol was only verified through phone interviews, therefore it is not possible to exclude that some participants did not perform all the training sessions. Fourth, outcomes were only based on validated questionnaires and scales, and objective measures of urogenital functions could provide additional information on telerehabilitation efficacy. Finally, a follow-up assessment could detect the duration of the symptoms’ improvement. However, this study provided early evidence of the feasibility and efficacy of telerehabilitation for urogenital disorders, suggesting that also a protocol based on pre-recorded videos could improve most of the reported symptoms. Future studies on larger samples and with subgroups based on symptom severity should be performed, using dedicated hardware and software to deliver the intervention with standardized technical characteristics, considering some objective measures of urogenital function, and comparing the proposed telerehabilitation protocols with a control group, as well as a group receiving a similar protocol in person.

## Conclusions

In summary, the results from the present study provide preliminary evidence of the improvements in urogenital symptoms after telerehabilitation. In particular, females with MS were found to improve most of the self-reported incontinence and sexual function outcomes, improving their QoL and depression symptoms. Despite both protocols being effective, live video sessions with a physiotherapist led to greater improvements than home-based video exercise. The choice of the telerehabilitation protocol should prefer the live video sessions, but pre-recorded videos could be considered to increase participation and reduce costs.

## Electronic supplementary material

Below is the link to the electronic supplementary material.

Supplementary Material 1

## Data Availability

Anonymized data can be requested upon reasonable request to the corresponding author.
