# Supplementary material for: Comparison of different telerehabilitation protocols for urogenital symptoms in females with multiple sclerosis: a randomized controlled trial
Source: Neurol Sci. 2024 Sep 3;45(11):5501–9. doi: 10.1007/s10072-024-07742-y (PMC11470853; doi:10.1007/s10072-024-07742-y)
Supplement: Supplementary file 1 — Supplementary Material 1 [file 10072_2024_7742_MOESM1_ESM.docx]

**Supplementary file 1**

The proposed telerehabilitation included a multidomain physiotherapy protocol which consisted of 45-minute sessions with increasing training intensity according to the participant's self-reported performance and fatigue. To support the evaluation of the perceived intensity, a 6-20 rate of perceived exertion (RPE) scale was suggested during the live video training, and the prerecorded videos also suggested using RPE to increase the exercise intensity. The training sessions provided to the two groups were similar in terms of contents and recommendations. During the live video training (REMOTE), the physiotherapist showed each exercise and immediately provided feedback on the participants’ execution. During the prerecorded video training (SELF), the exercise execution was clearly shown but no feedback to the participant was possible.

The contents of the training sessions were:

1. Breathing exercises:

The physical therapy session started with breathing exercises. The efficacy of breathing exercises for urogynecological problems is still debated [23]. Breathing exercises have been suggested to help the patient to relax and concentrate, as well as it has been suggested being important to promote proper functioning of the pelvic floor muscles and control over the bladder [24]. In addition, deep breathing can help in reducing anxiety and improving body awareness during sexual activity in men [30]. Duration of the exercises: approximately 5 minutes. According to previous literature [26], the proposed exercises were:
- diaphragmatic breathing, lying, sitting and standing, 30 repetitions × 3 sets.
- in the last 4 training sessions, functional expiratory patterns were performed, including nose blowing, coughing, laughing and sneezing.

1. Pelvic Floor Muscle Training (PFMT):

PFMT represents the core activity for bladder and sexual dysfunctions, also as a telerehabilitation protocol in Multiple Sclerosis [8, 25]. The protocol was based on previous literature [27], and started with a progression of exercises selected with a specific goal: improving the proprioception and the control of the pelvic floor muscles. First, exercises were focused on the control of the activation of the pelvic floor muscles without the activation of other synergic muscles (glutei, abductors, and abdominal muscles). Then, a progression of pelvic floor exercises was included from the modified gynecological position to the standing position. Finally, pelvic floor exercises were presented with dynamic tasks such as dead bug, bird dog and squat. To phasic and tonic muscles, all exercises presented were modulated with rapid contractions with resting phases or with slow and maintained contractions. Before each section, subjects were asked to void the bladder. Duration of the exercises: approximately 20 minutes.

1. Proprioceptive exercises:

Proprioceptive exercises aim to improve the ability to control the position and movement of a body part in space by also enhancing the awareness of one’s own body. Proprioceptive neuromuscular facilitation (PNF) has been suggested could be used to increase pelvic floor muscles’ recruitment in young healthy women [28]. During the physiotherapy sessions, exercises were focused on general proprioception and the specific pelvic floor proprioception, implementing some exercises from the PNF-concept (i.e., anterior elevation, posterior depression, anterior depression, and posterior elevation) [28]. The physiotherapist invited patients to focus on sensations coming from their pelvic floor. Duration of the exercises: approximately 5 minutes.

1. Motor coordination and balance:

Motor coordination and balance impairment in multiple sclerosis are associated with a lack of trunk control. Core stability training could stabilize the lumbar and pelvis area and improve balance and mobility [31]. To improve balance and motor control core stability activation was associated with balance exercises. Duration of the exercises: approximately 5 minutes. Participants were asked to:

- tread from foot to foot with concomitant activation of core stability muscles.
- stand up and sit on a chair with concomitant activation of core stability muscles.
- step to the left, to the right, forward, backward with concomitant activation of core stability muscles.

1. Functional training:

In the last 4 sessions of the training, the participants were invited to perform motor tasks that are usually part of the typical daily activities, with a focus on pelvic floor muscles control. These exercises were performed to translate the muscle control exercises that were trained in the prior training, into more complex and practical activities, to improve. Duration of the exercises: approximately 5 minutes.

1. Body scan technique:

To increase the attention to bodily sensations, body scan techniques were included. Participants were asked to find a quiet place without smartphones, television and radio to find a comfortable position and to stay with their eyes closed. A 20-minute body scan through the entire body from the foot to the head was performed. The body scan exercise started with a mental visualization of the foot, followed by a mental visualization of the knee and hips. Then the mental visualization was addressed to the trunk, and the upper limbs, finally to the head [29]. Duration of the exercises: approximately 5 minutes.
